# Supplementary material for: Spin Crossover and Thermochromism in Iron(II) Complexes with 2,6-Bis(1H-imidazol-2-yl)-4-methoxypyridine
Source: Int J Mol Sci. 2023 Jun 7;24(12):9853. doi: 10.3390/ijms24129853 (PMC10298036; doi:10.3390/ijms24129853)
Supplement: Supplementary file 1 [file ijms-24-09853-s001.zip › ijms-2429614-supplementary.pdf]

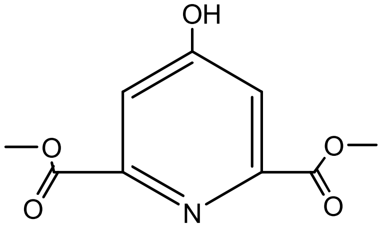

1

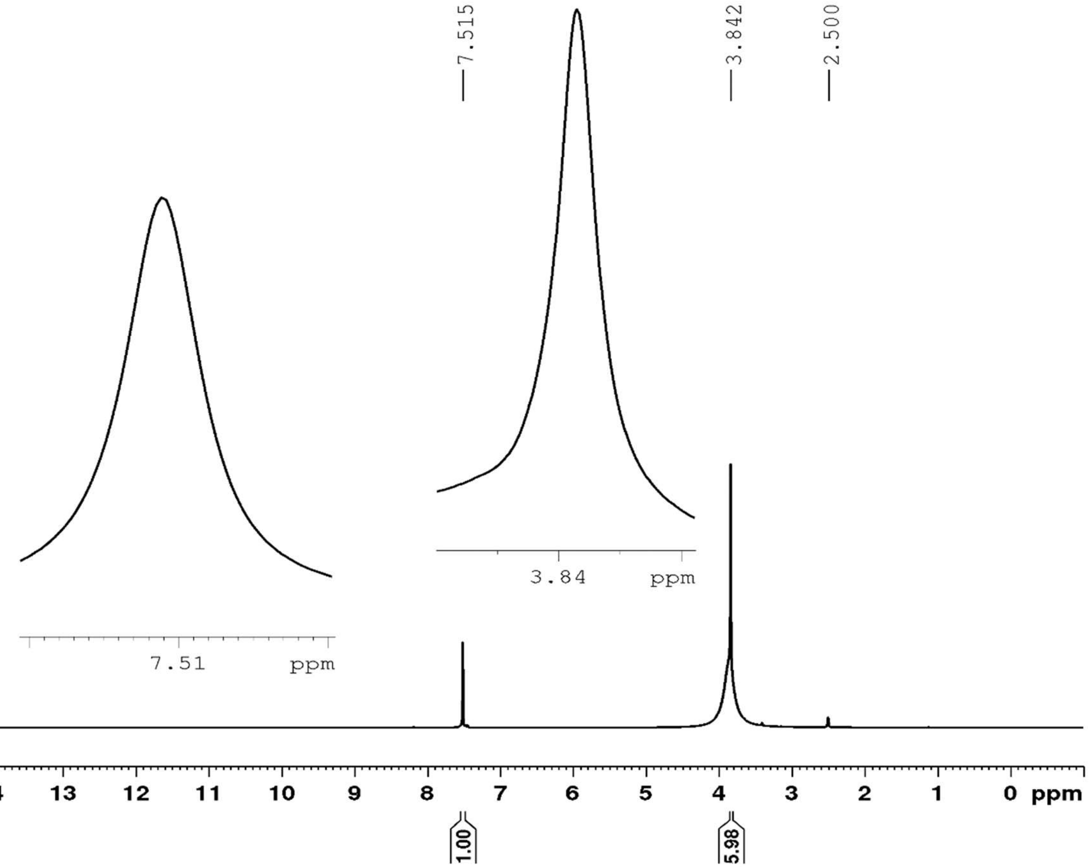

Figure S1. ^{1}H NMR spectrum of **1**, AV 400, NS 8, DMSO-d_6.

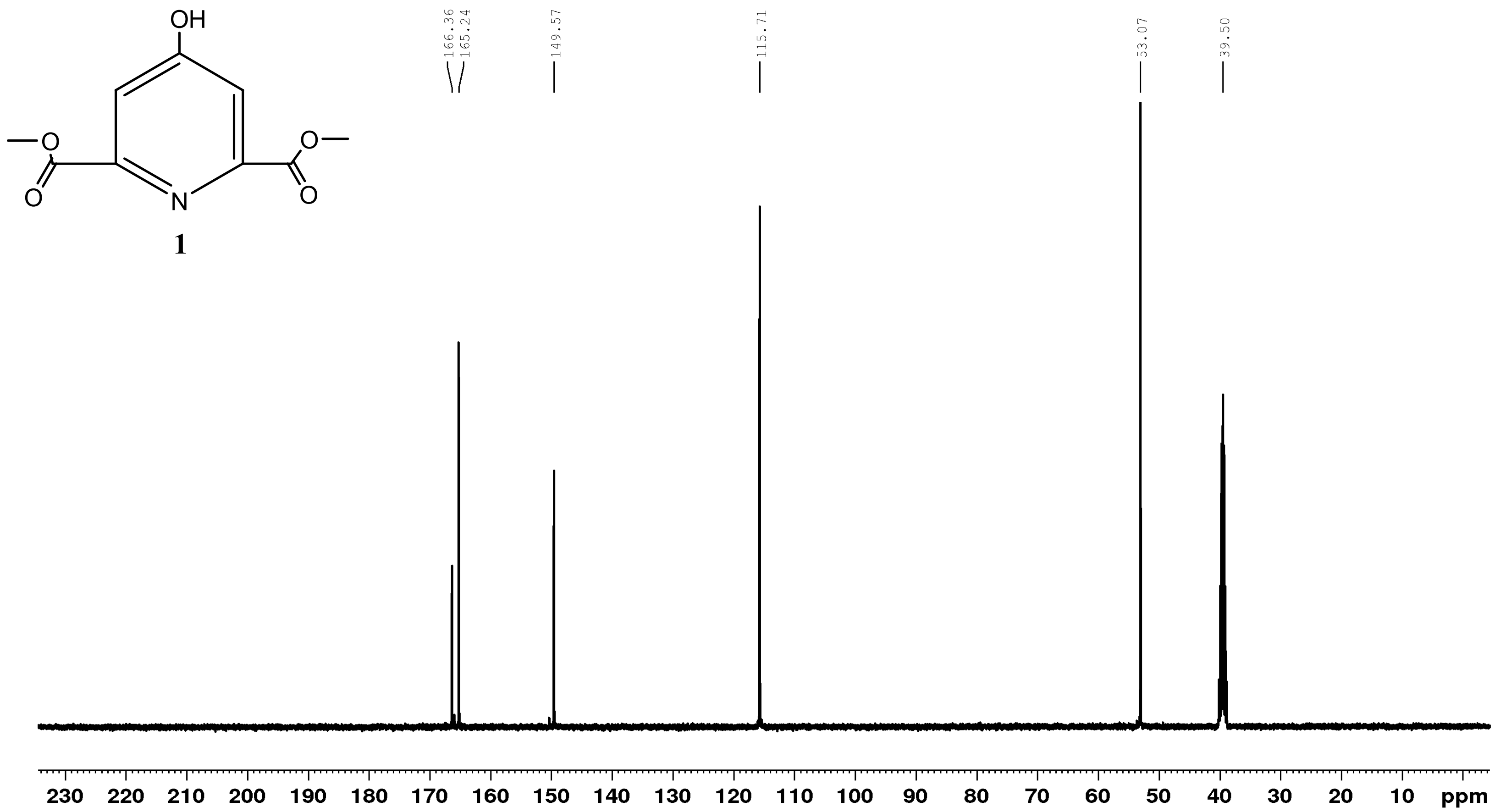

1

Figure S2. ^{13}C NMR spectrum of **1**, AV 400, NS 208, DMSO-d_6.

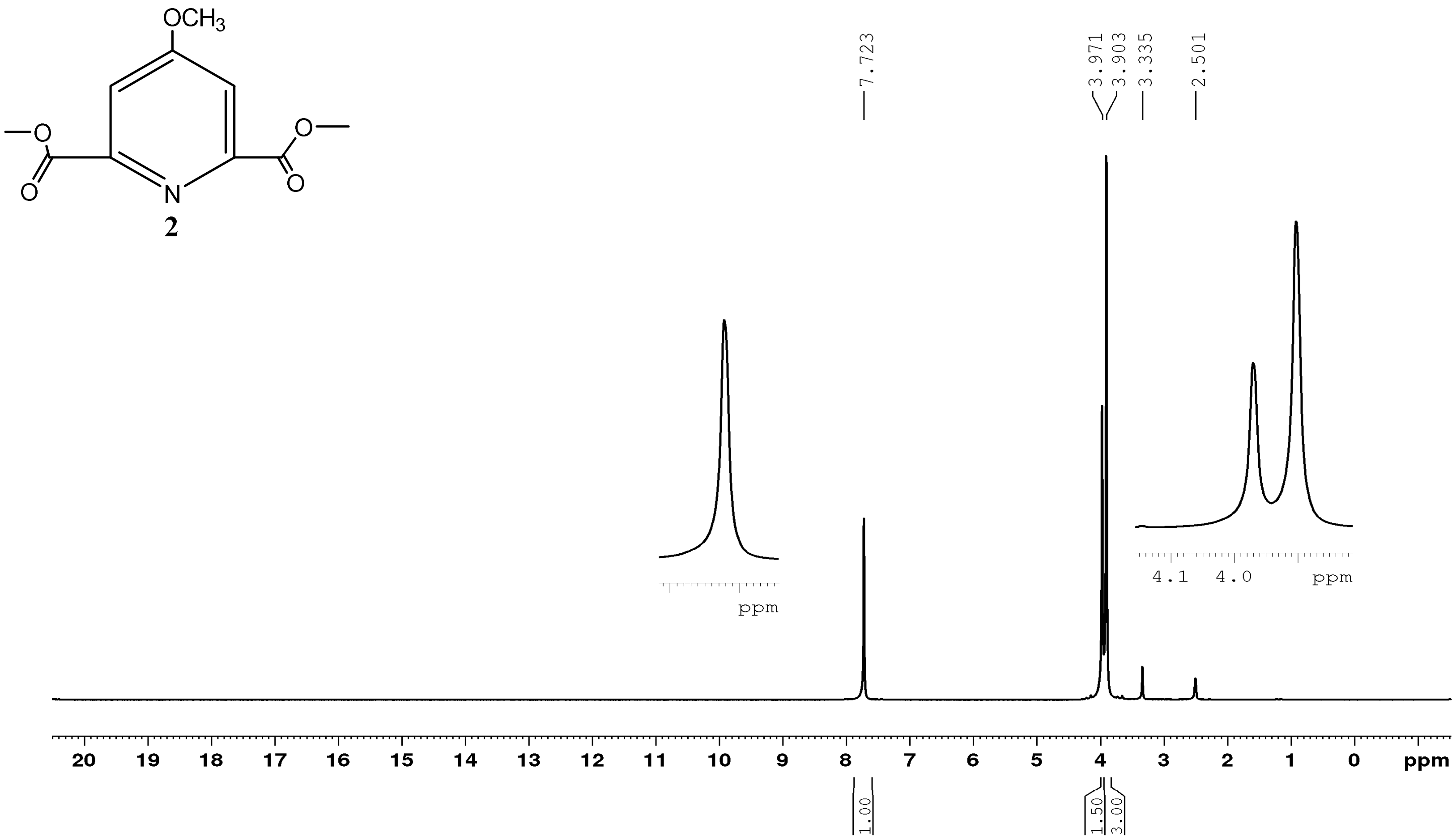

Figure S3. ^{1}H NMR spectrum of **2**, AV 300, NS 16, DMSO-d_6.

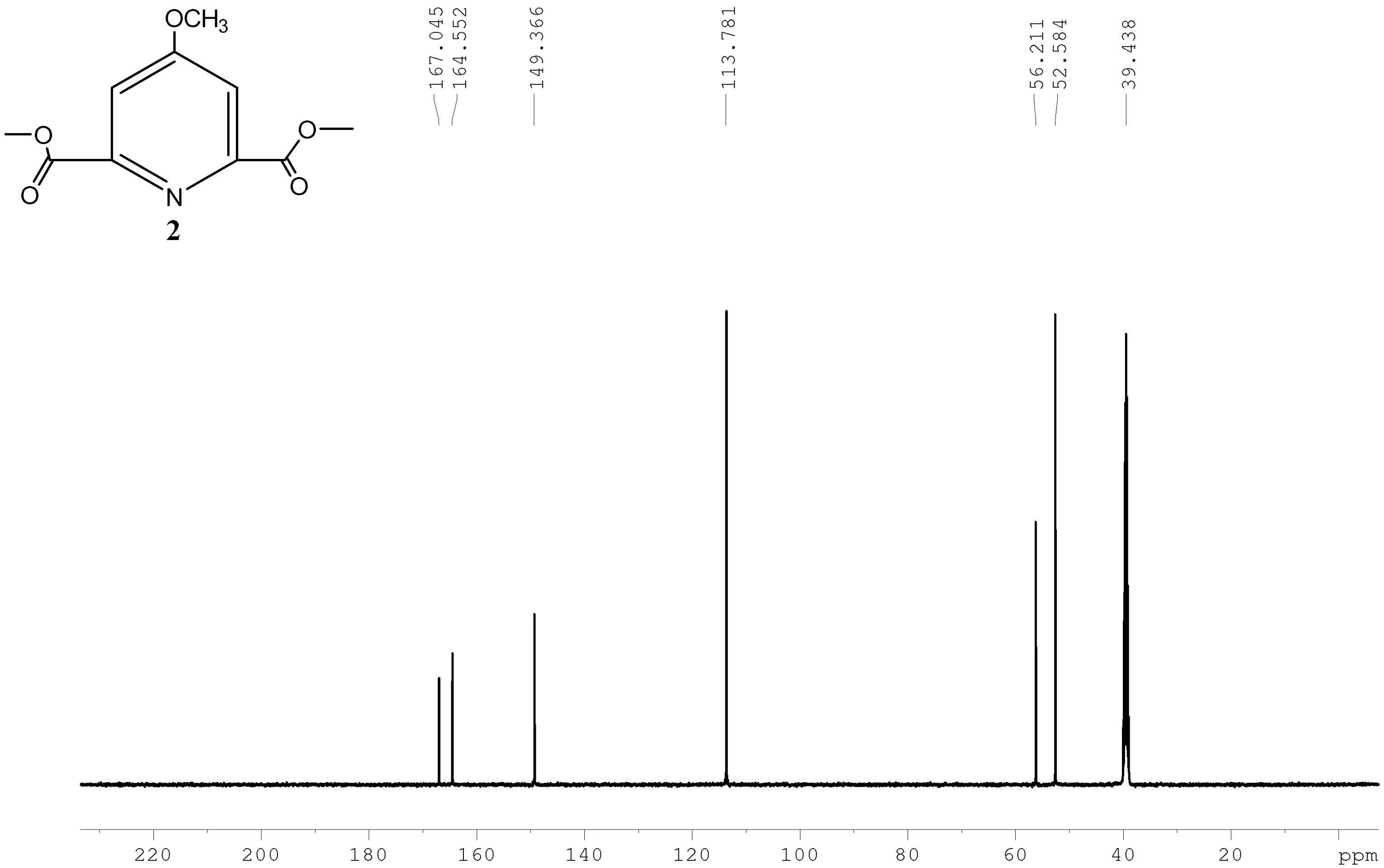

Figure S4. ^{13}C NMR spectrum of **2**, AV 500, NS 672, DMSO-d_6.

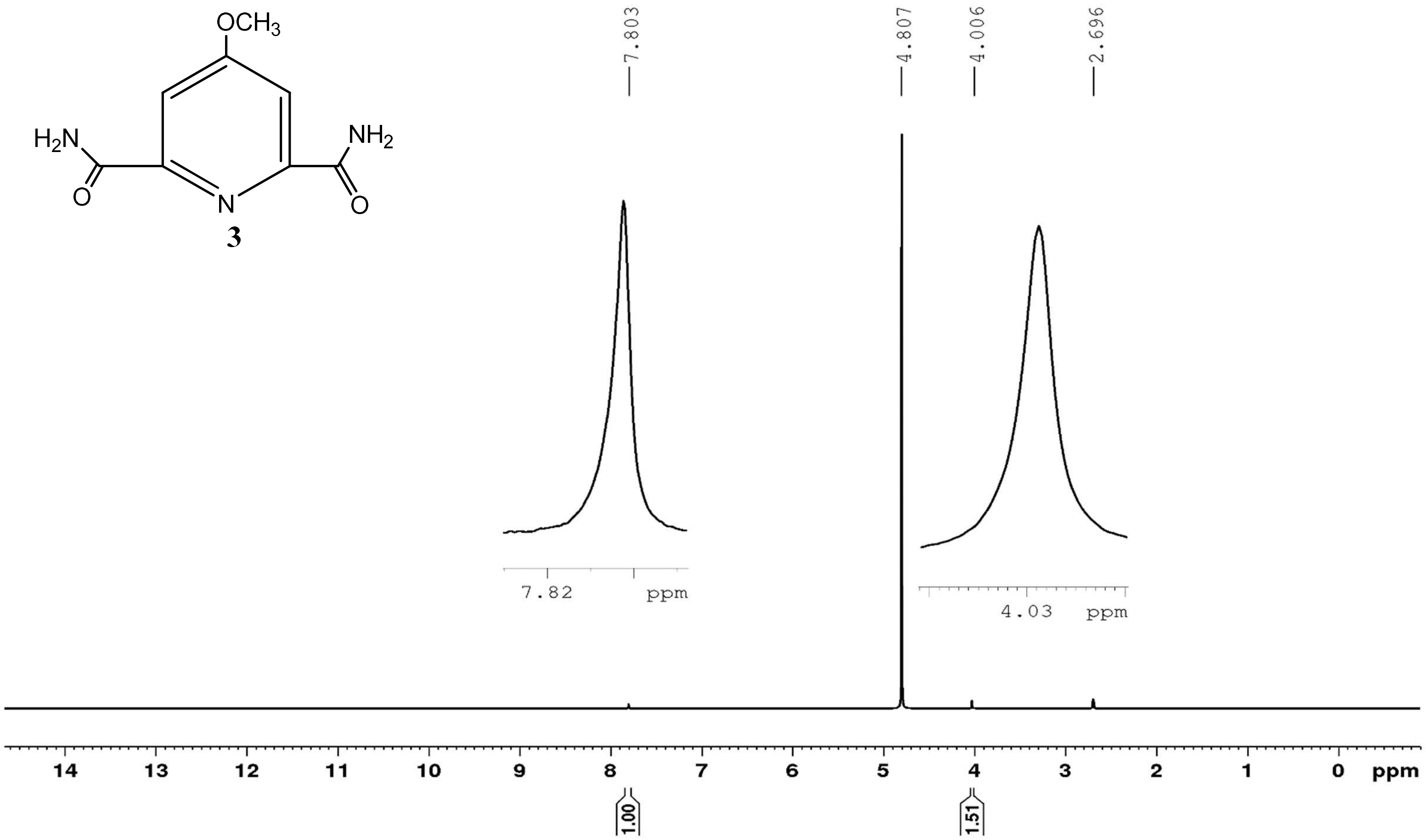

Figure S5. ^{1}H NMR spectrum of **3**, AV 300, NS 16, D_2O/DMSO-d_6.

.

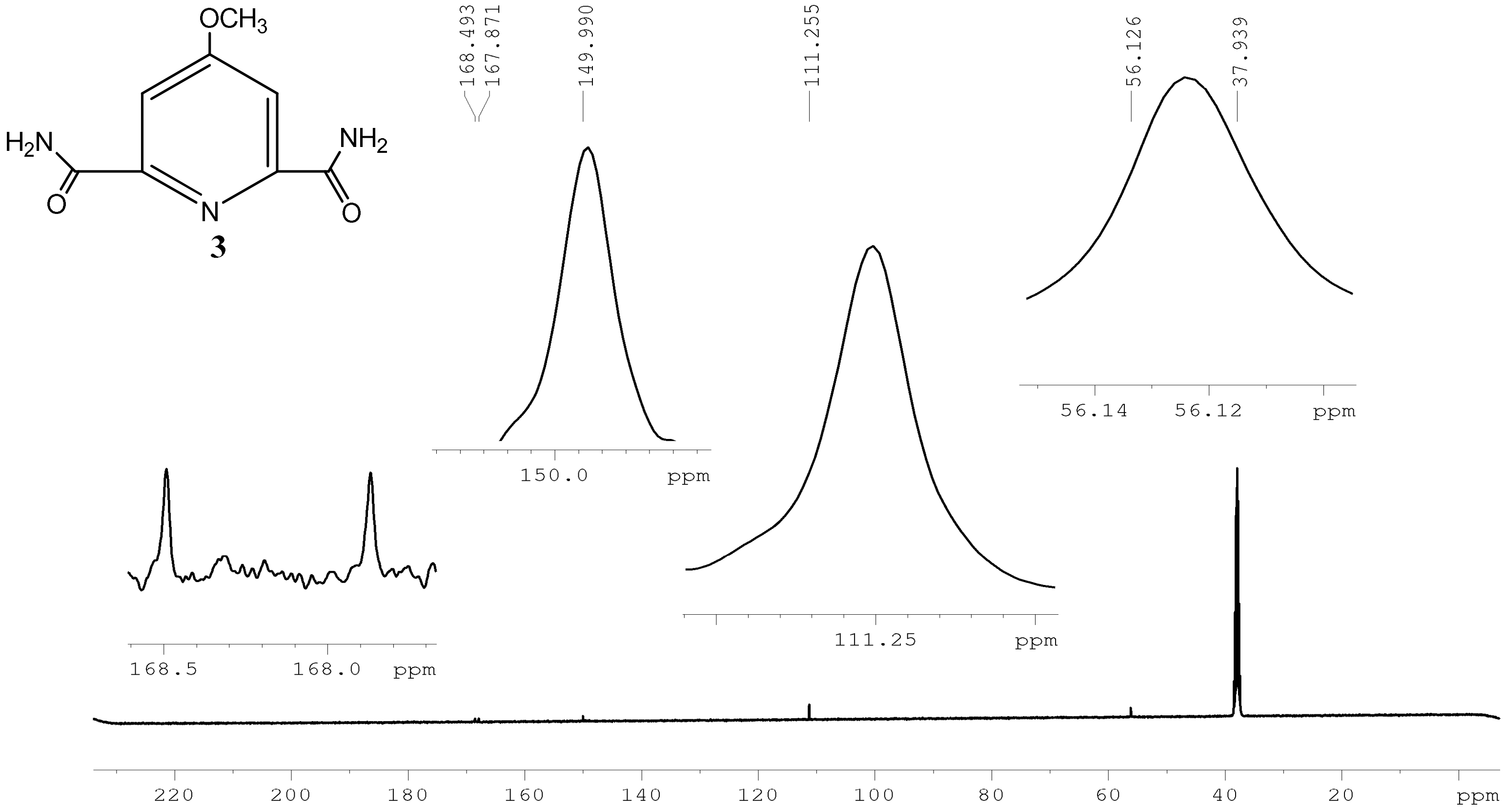

Figure S6. ^{13}C NMR spectrum of **3**, DRX 500, NS 30944, D_2O/DMSO-d_6.

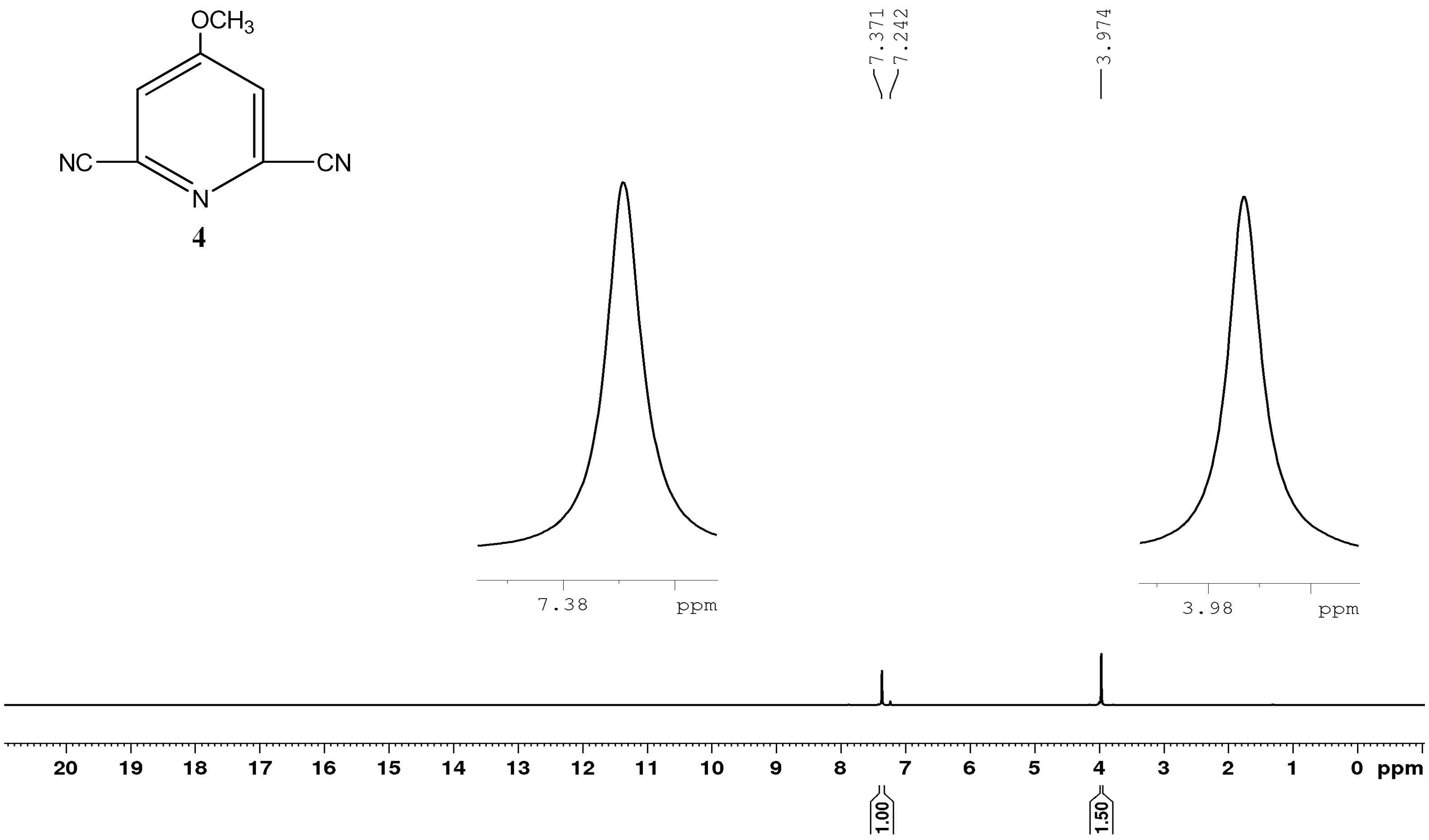

Figure S7. ^{1}H NMR spectrum of **4**, AV 400, NS 16, $CDCl_3$.

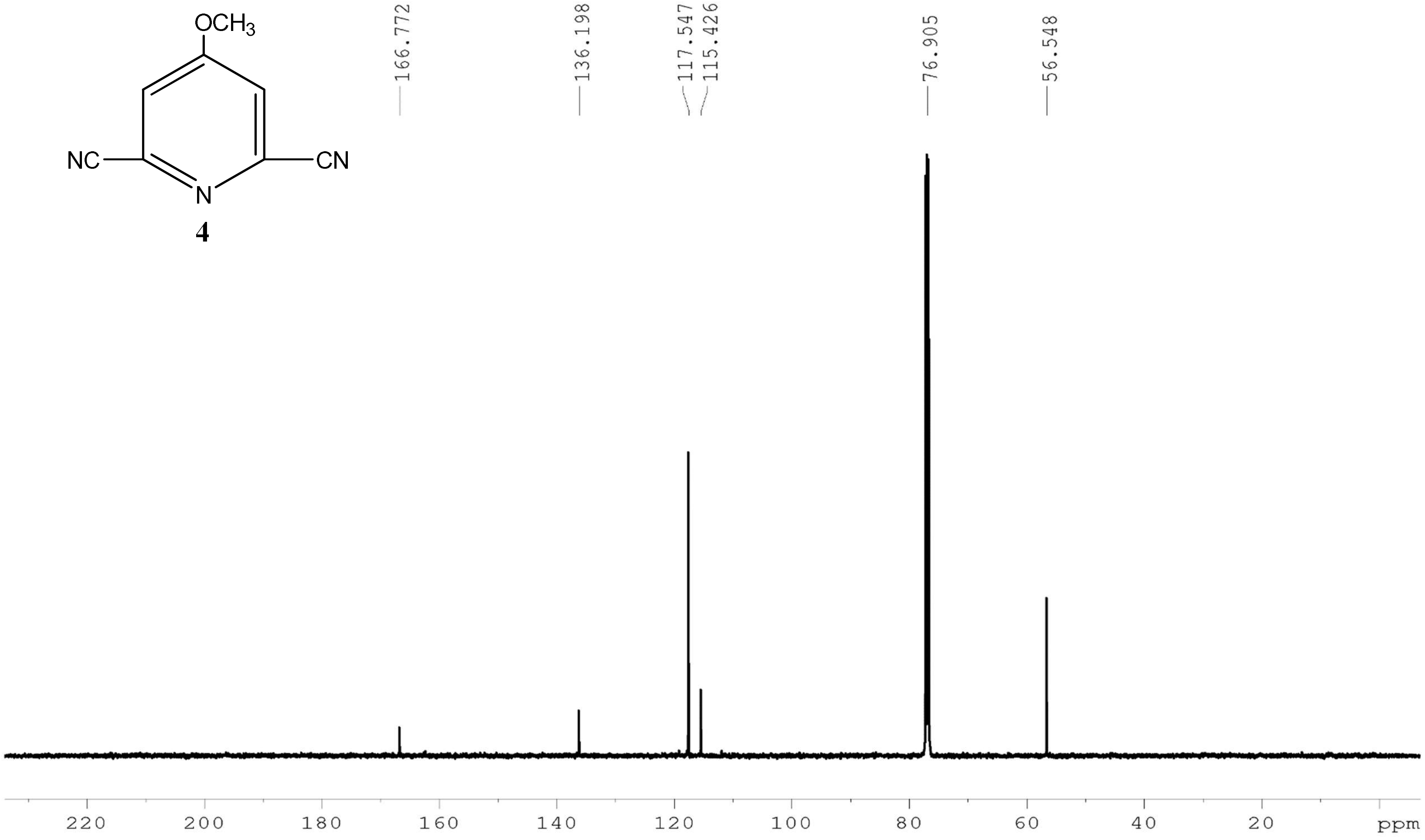

Figure S8. ^{13}C NMR spectrum of **4**, DRX 500, NS 2304, $CDCl_3$.

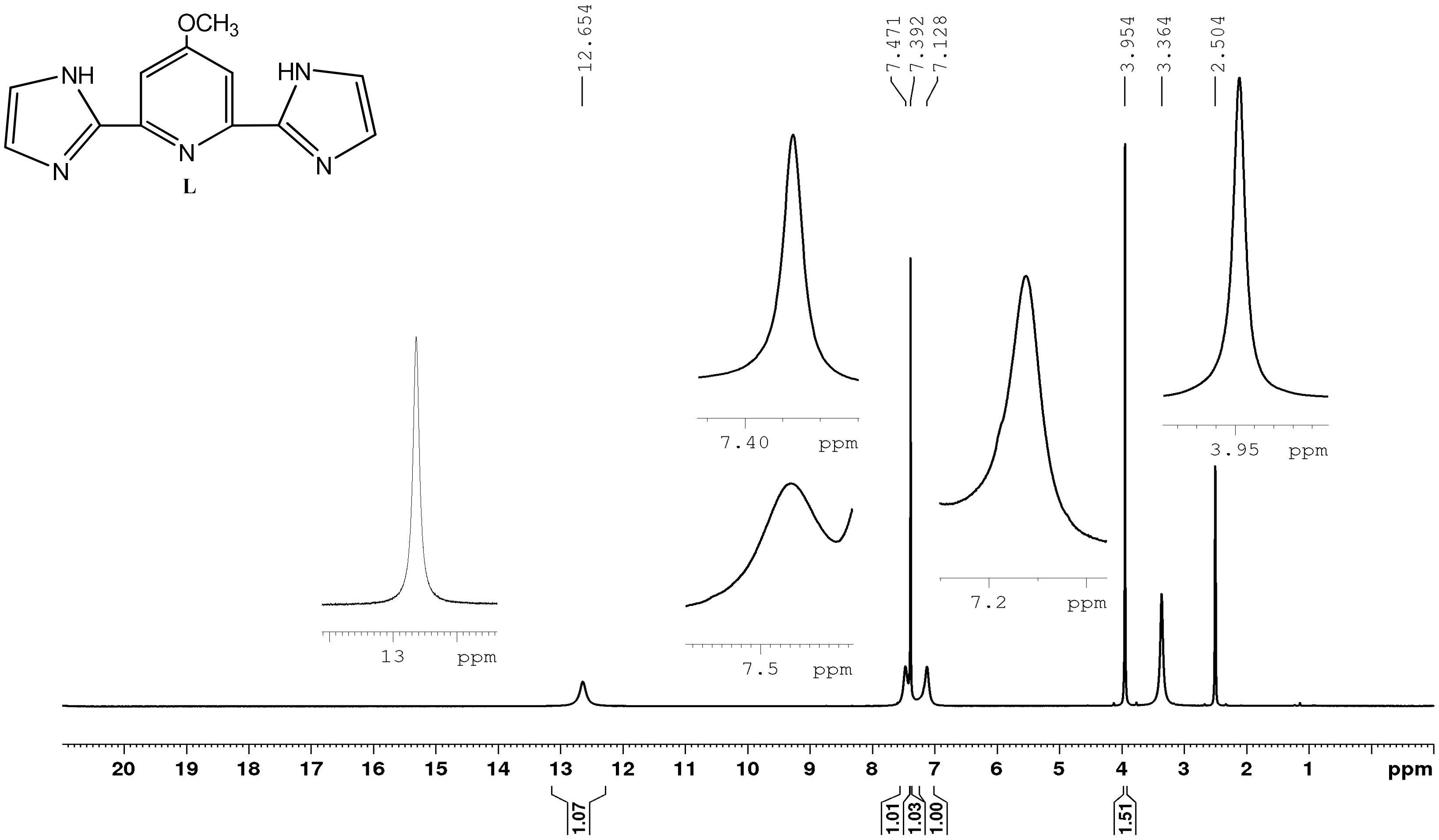

Figure S9. ^{1}H NMR spectrum of **L**, AV 400, NS 80, DMSO-d6.

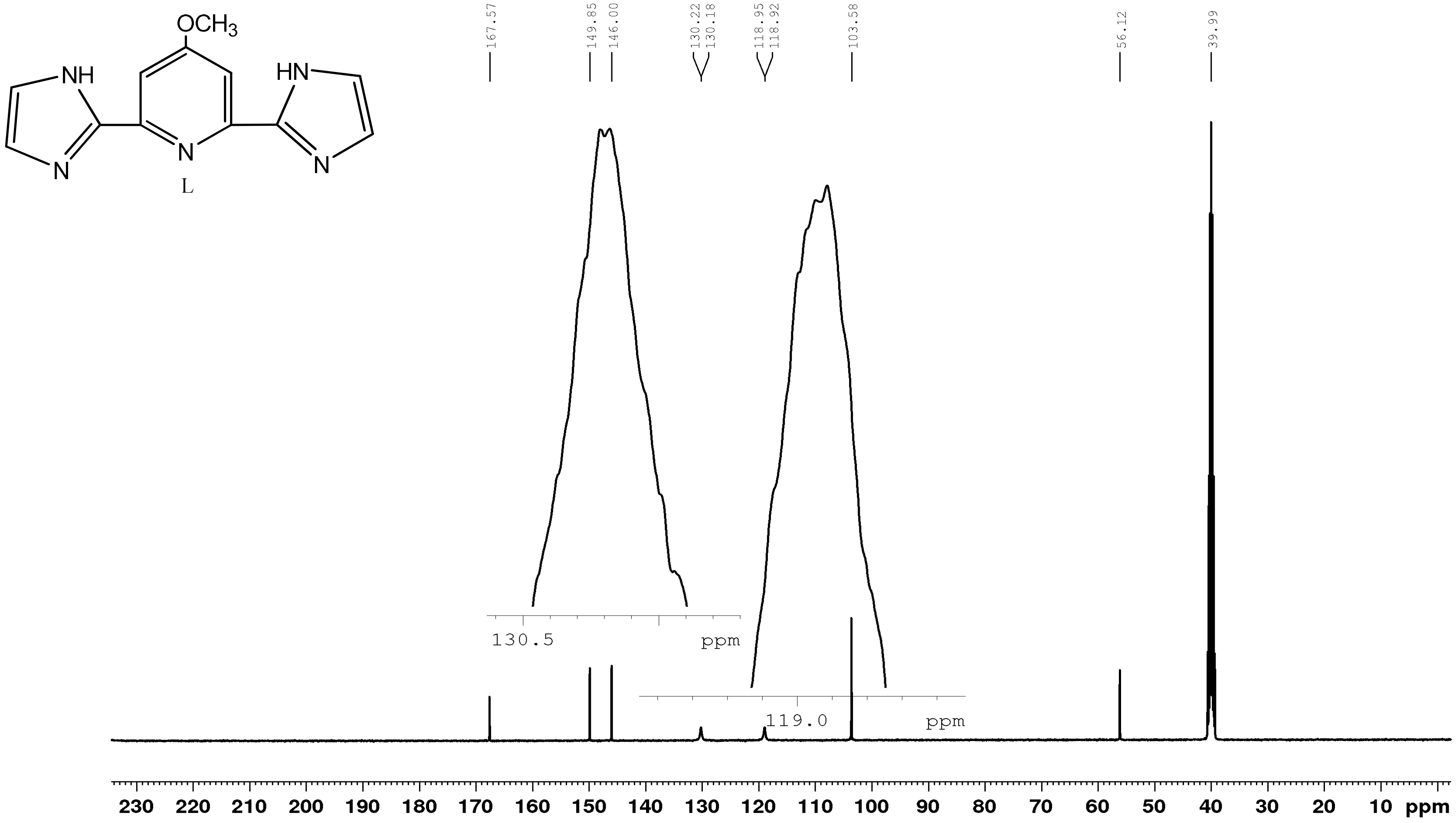

Figure S10. ^{13}C NMR spectrum of **L**, AV 400, NS 6832, DMSO-d_6.

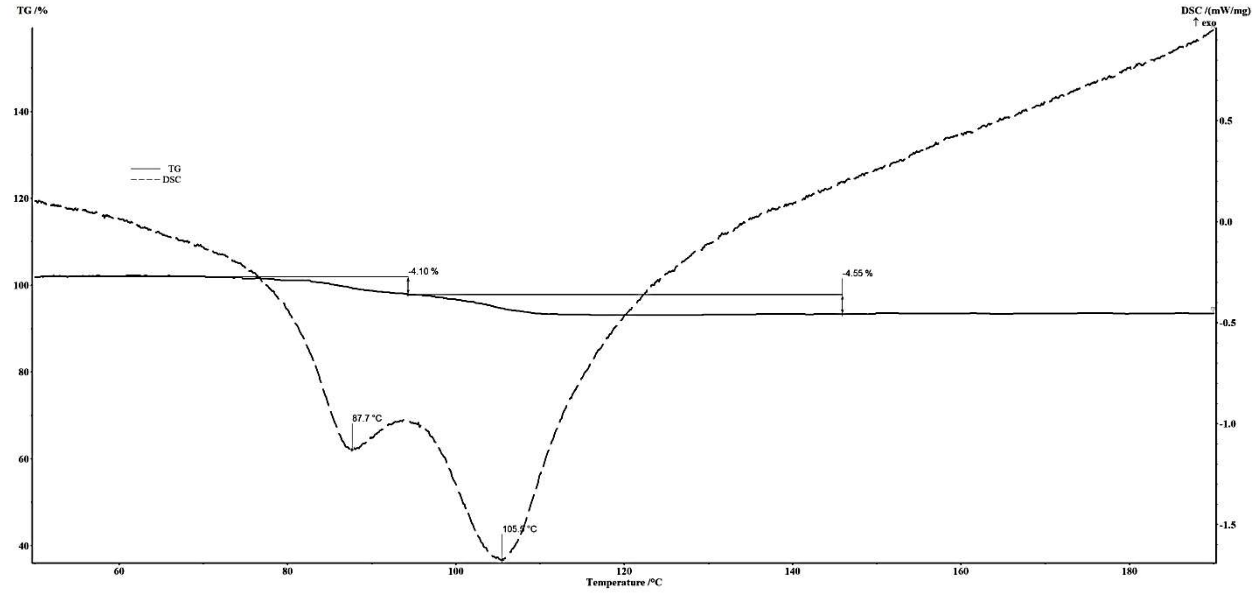

Figure S11. TG/DSC curves of **L·1.2H$_2$O**

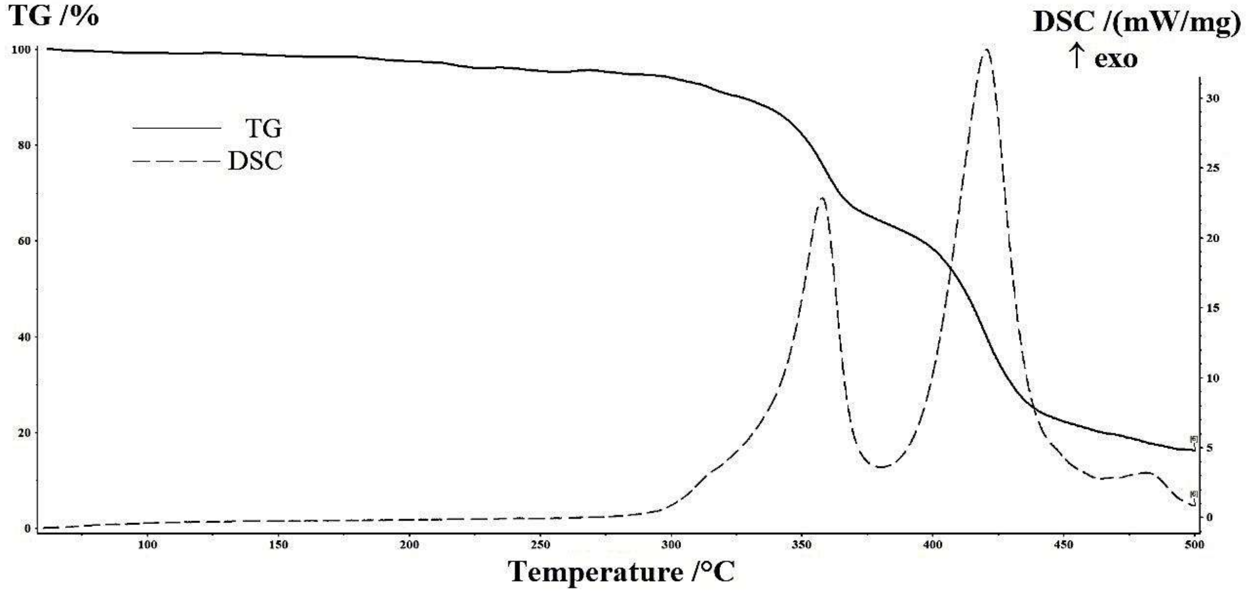

Figure S12. TG/DSC curves of **I**

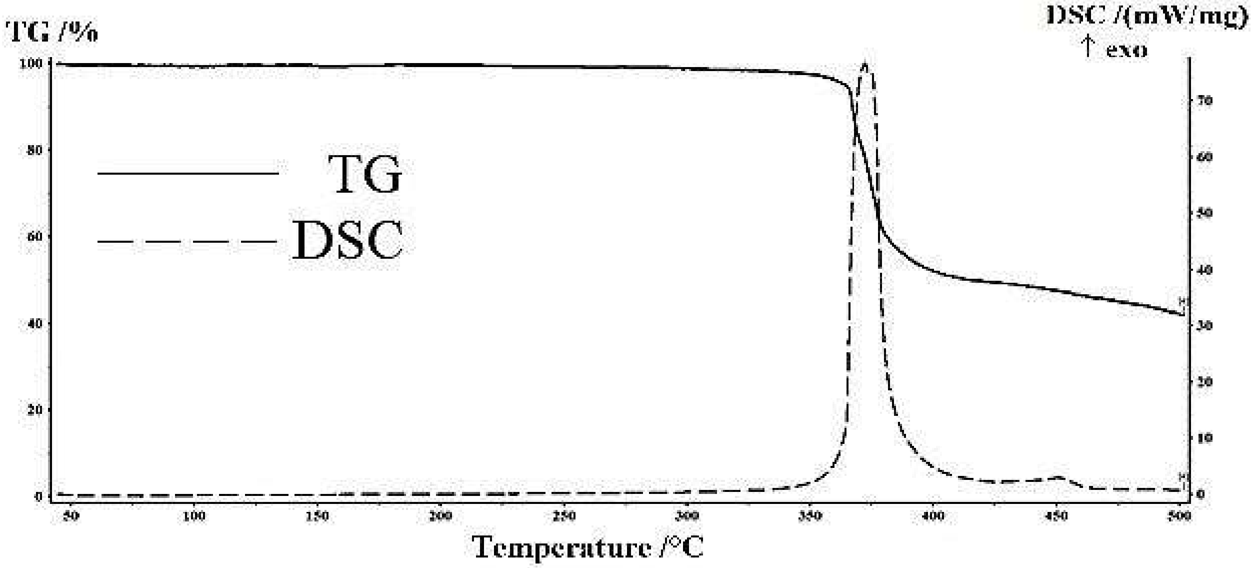

Figure S13. TG/DSC curves of **II**

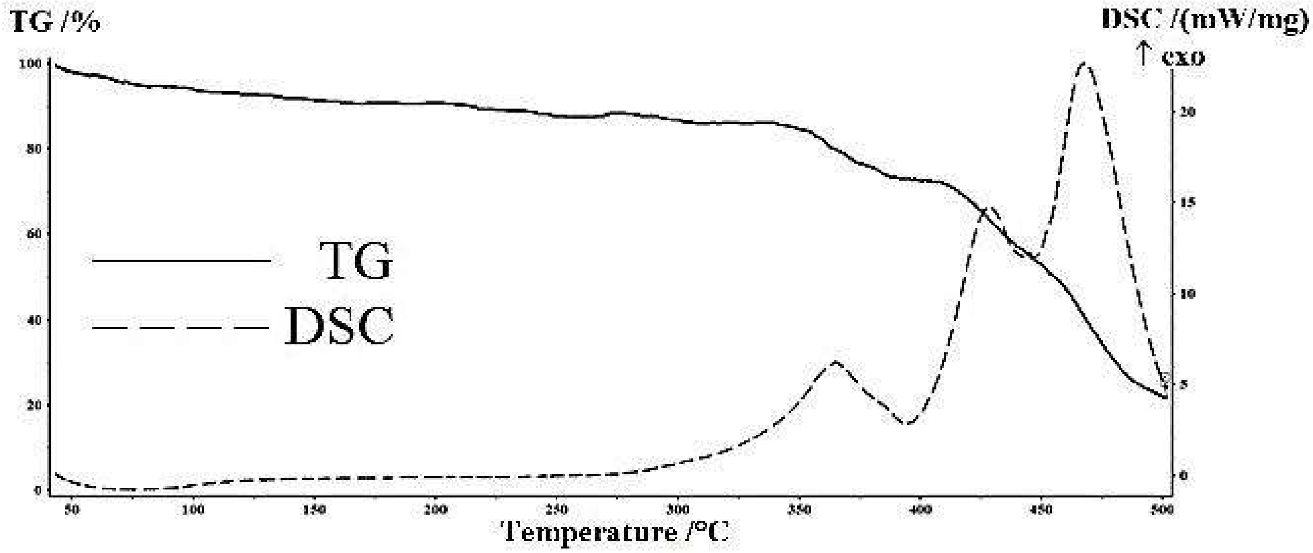

Figure S14. TG/DSC curves of **III**

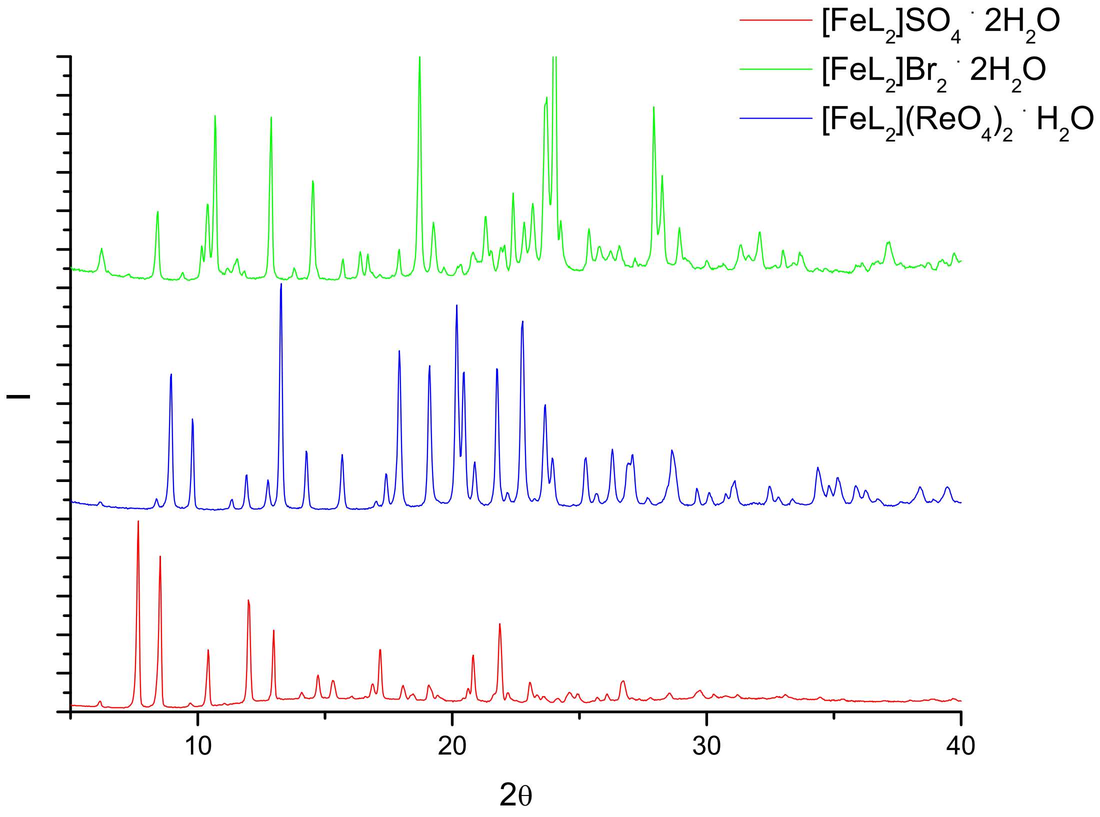

Figure S15. Diffractograms of complexes

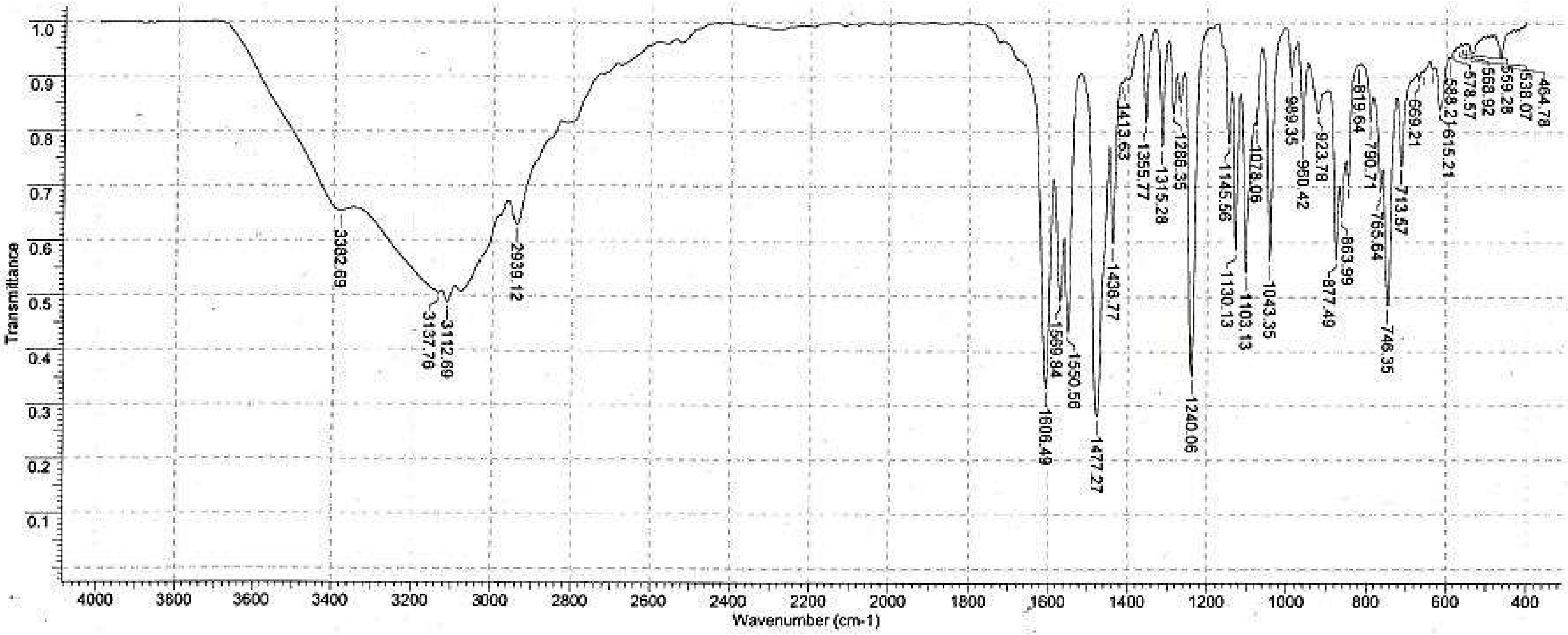

Figure S16. IR spectrum of **L**

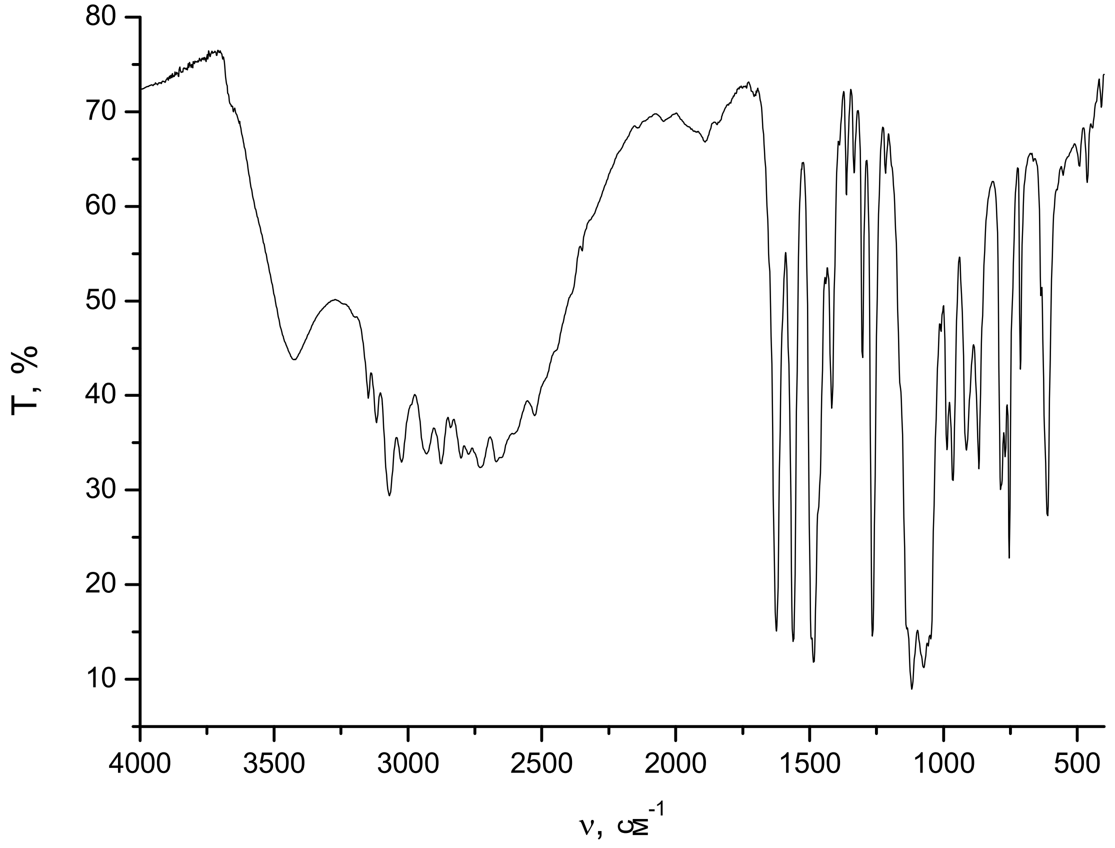

Figure S17. IR spectrum of **I**

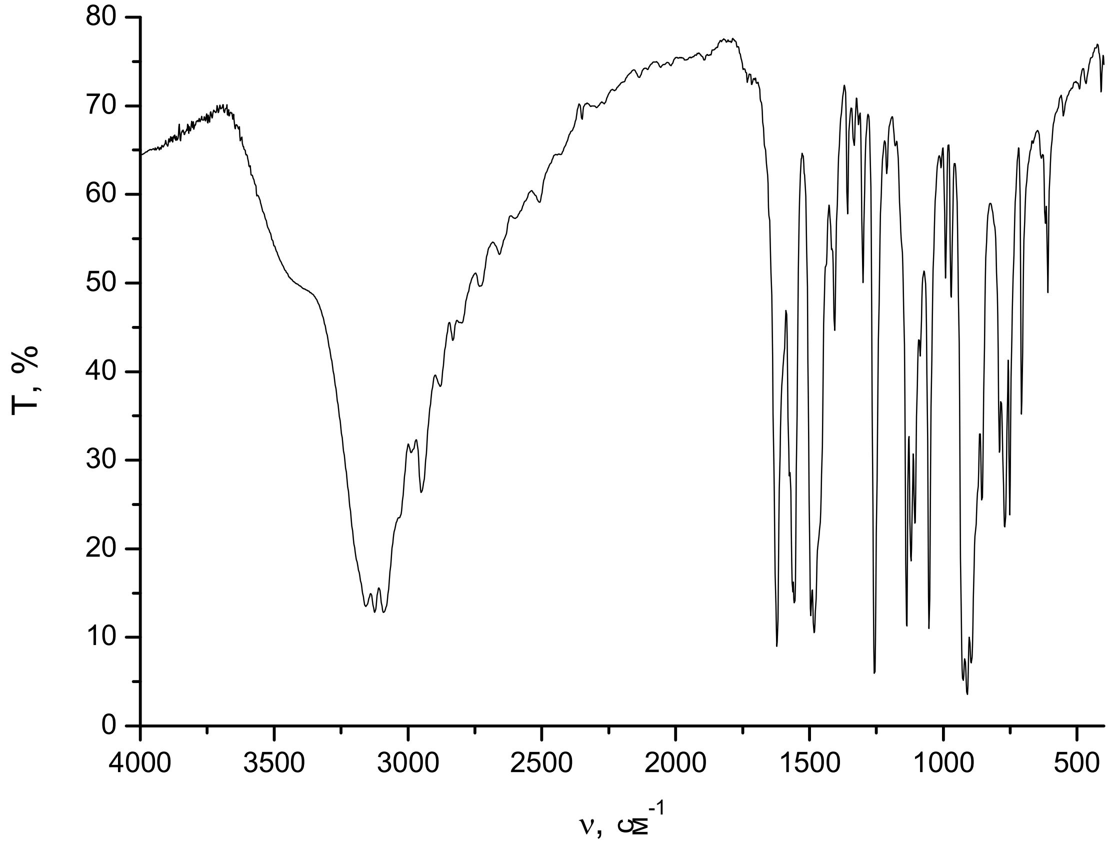

Figure S18. IR spectrum of **II**

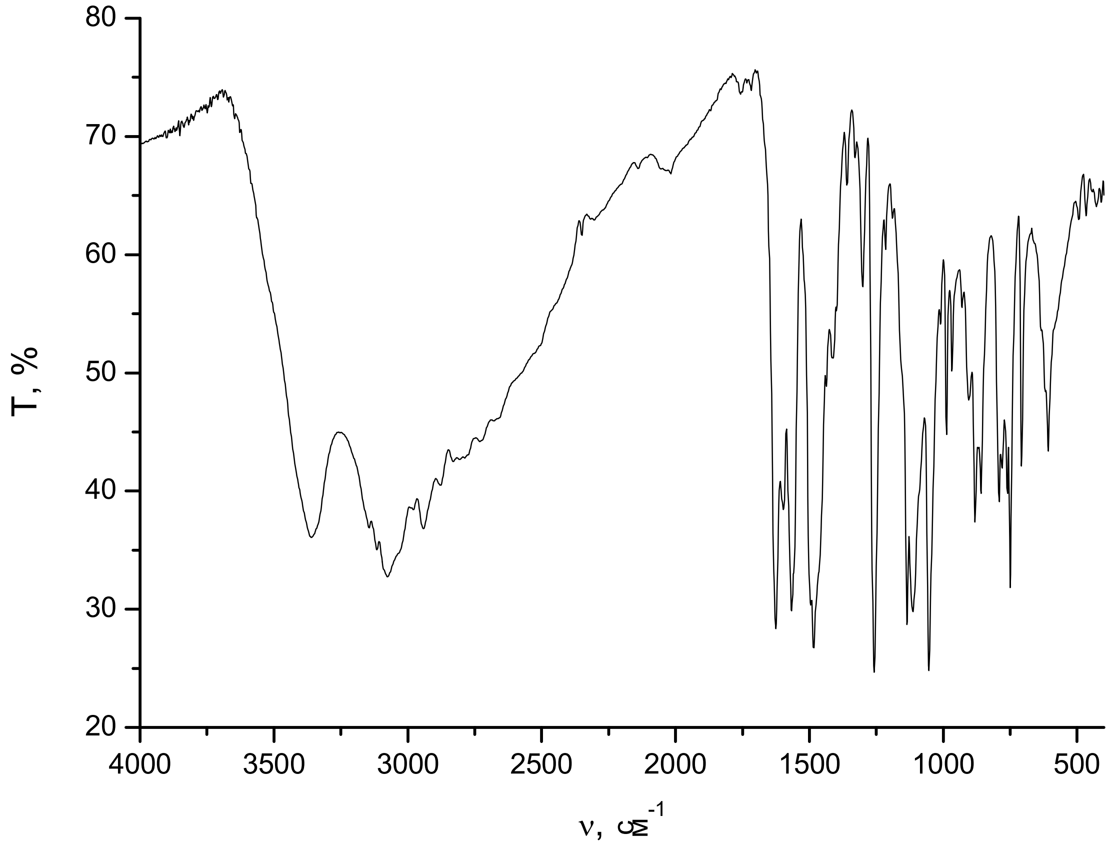

Figure S19. IR spectrum of **III**

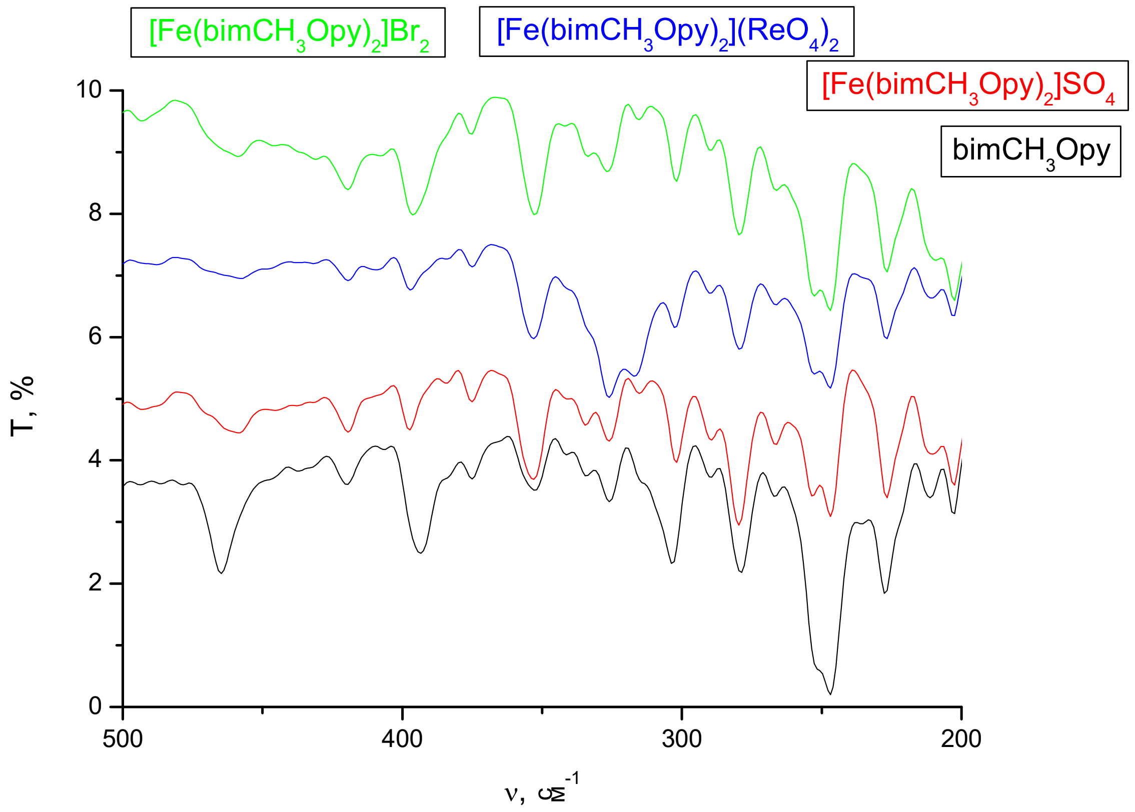

Figure S20. Low-frequency IR spectra of **L** and **I** – **III**

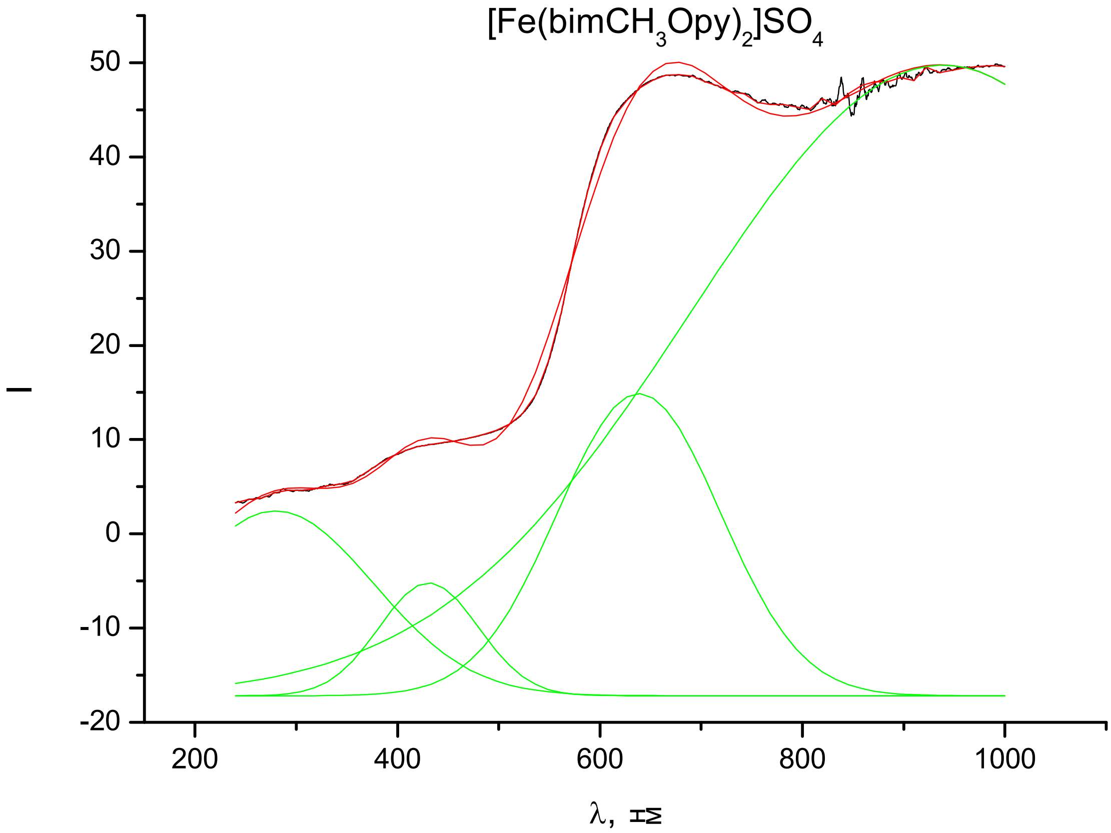

Figure S21. DRS of **I**

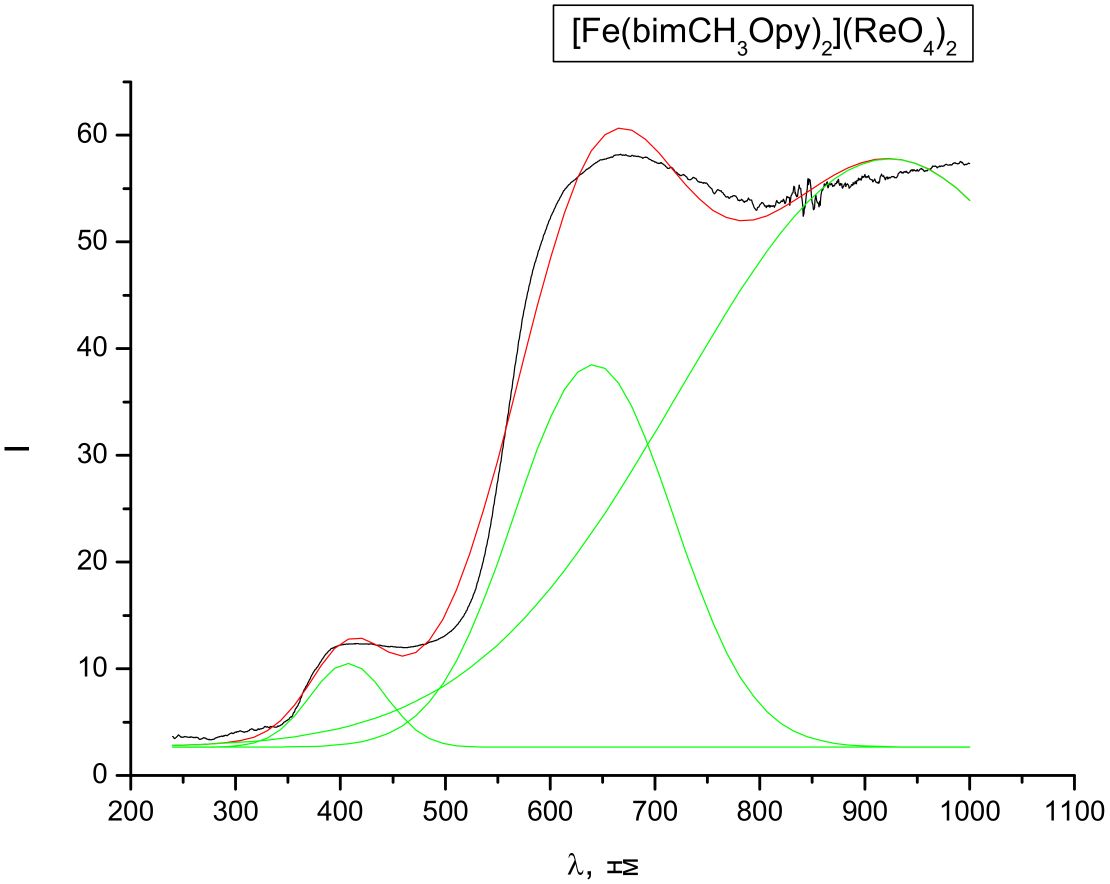

Figure S22. DRS of **II**

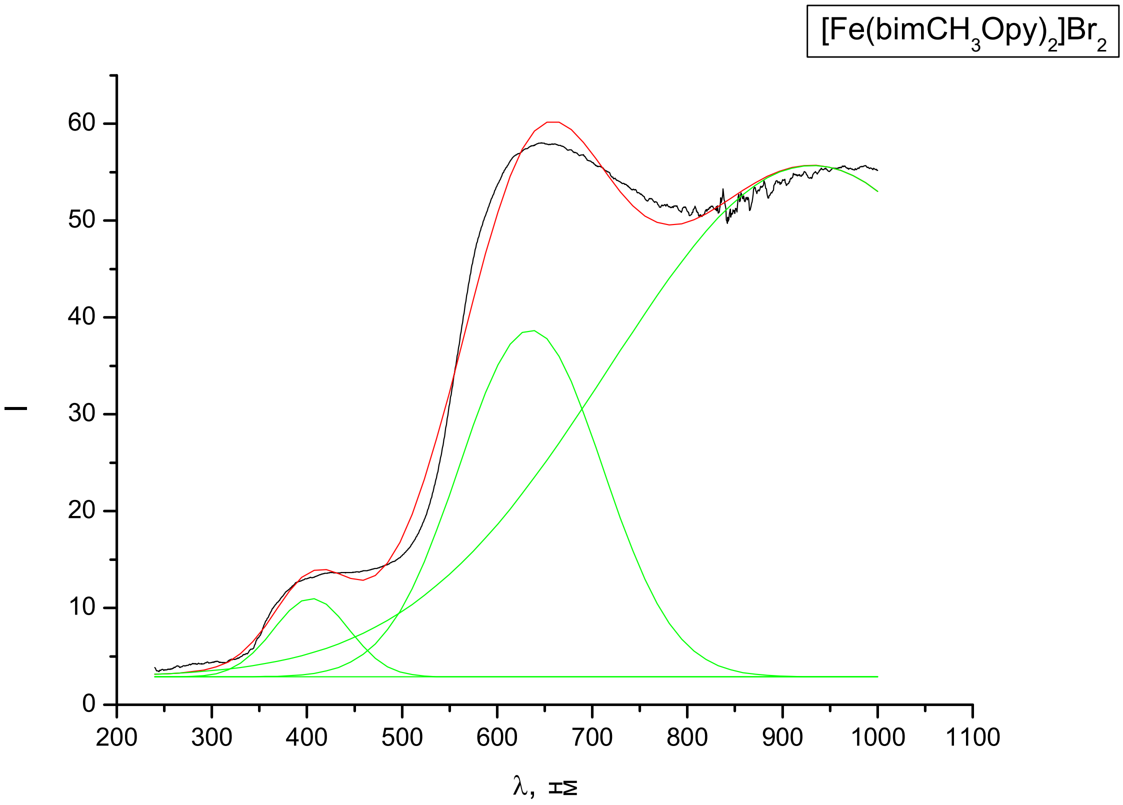

Figure S23. DRS of **III**
